# Supplementary material for: Reaction-Based Amine and Alcohol Gases Detection with Triazine Ionic Liquid Materials
Source: Molecules. 2019 Dec 27;25(1):104. doi: 10.3390/molecules25010104 (PMC6982797; doi:10.3390/molecules25010104)
Supplement: Supplementary file 1 [file molecules-25-00104-s001.pdf]

Reaction-Based Amine and Alcohol Gases Detection with Triazine Ionic Liquid Materials

Hsin-Yi Li and Yen-Ho Chu*

Department of Chemistry and Biochemistry, National Chung Cheng University, 168 University Road, Minhsiung, Chiayi 62102, Taiwan, Republic of China

* Corresponding author. Tel: 886 52729139; fax: 886 52721040; e-mail: cheyhc@ccu.edu.tw

Content

Figure S1 3

Figure S2 4

Figure S3 6

Figure S4 8

^{1}H and ^{13}C NMR spectra, and HR-MS spectra 10

QCM Measurements 19

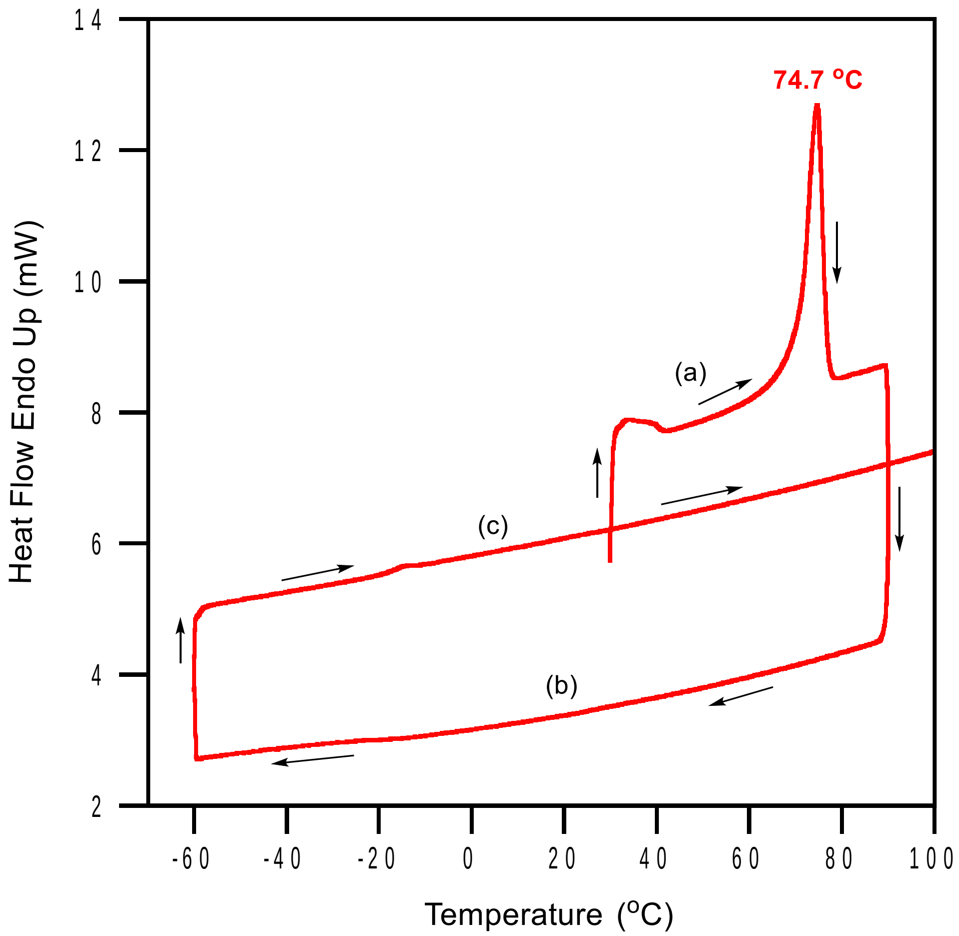

Figure S1. Differential scanning calorimetry (DSC) measurement for **AIL 1**, using a standard heat-cool-reheat (a→b→c) method, was performed on the DSC8500 instrument from PerkinElmer (Waltham, MA, USA). DSC data were collected in sealed aluminum pans with a cooling and heating rate of 10 °C/min. The result clearly showed that, upon first heating (a), an endothermic melting of **AIL 1** at 74.7 °C was observed and, on subsequent cooling (b), no detectable exothermic freezing point was obtained. For the same sample, the reheating (c) of **AIL 1** gave no more observable melting point, which demonstrated typically a supercooling property of **AIL 1**.

Figure S2. ESI-HRMS spectrum of reaction adduct of **AIL 1** with isobutylamine gas

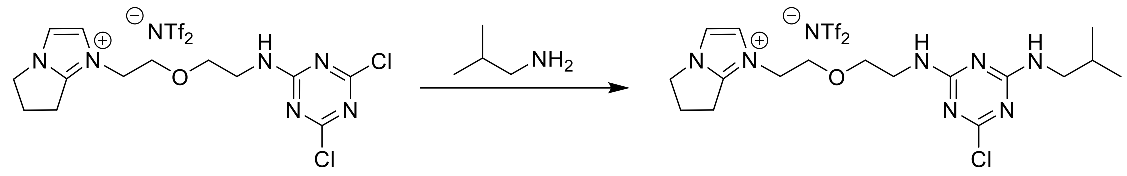

ESI-HRMS m/z $[M]^+$ calcd for $C_{17}H_{27}ClN_7O$ 380.1960, found 380.1950 ($[M]^+$, 100%), 382.1926 ($[M+2]^+$, 34%).

Spectrum

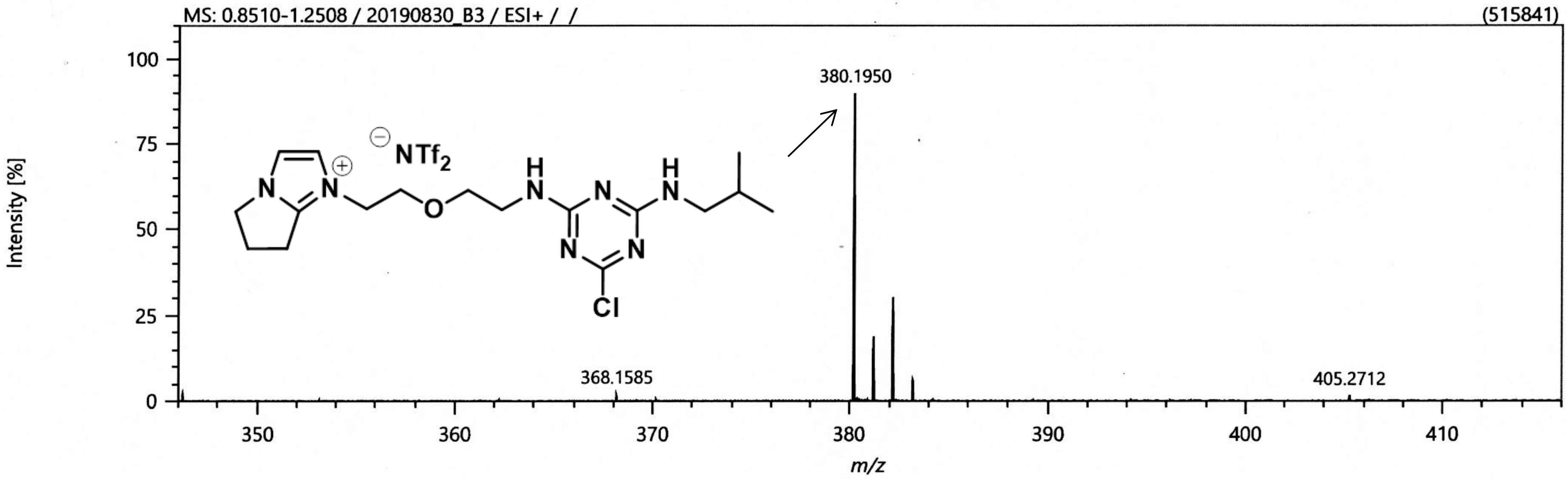

Elemental Composition

Parameters

Tolerance:	±100.00 ppm
Electron:	Odd/Even
Charge:	+1
DBE:	-1.5 - 999.0

Elements Set 1:

Symbol	C	H	O	Na	N	Si	Cl	S
Min	0	0	1	0	7	0	1	0
Max	400	1000	1	0	7	0	1	0

Results

Mass	Formula	Calculated Mass	Mass Difference [mDa]	Mass Difference [ppm]	DBE
380.19504	C17 H27 N7 O Cl	380.19601	-0.97	-2.55	7.5

Figure S3. ESI-HRMS spectrum of reaction adduct of **AIL 1** with propylamine gas

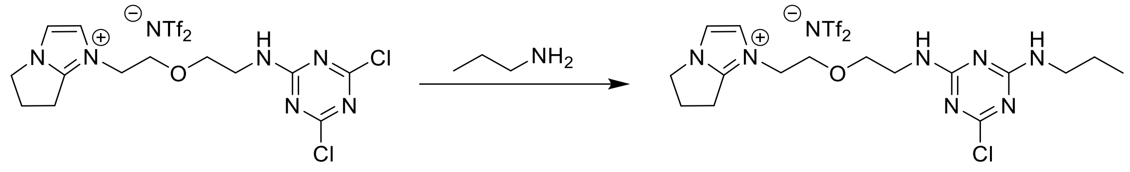

ESI-HRMS m/z $[M]^+$ calcd for $C_{16}H_{25}ClN_7O$ 366.1809, found 366.1814 ($[M]^+$, 100%), 368.1771 ($[M+2]^+$, 29%).

Elemental Composition Report

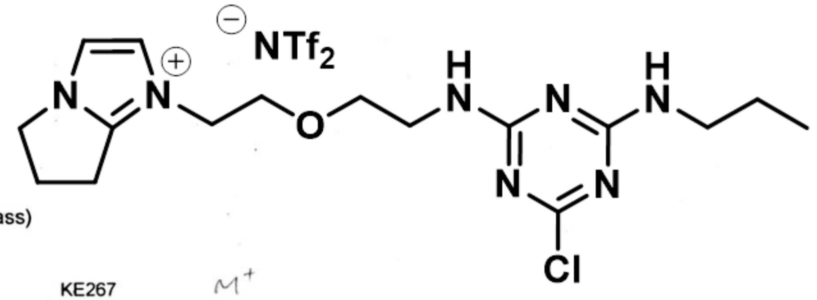

Multiple Mass Analysis: 2 mass(es) processed
Tolerance = 30.0 PPM / DBE: min = -1000.0, max = 1000.0
Element prediction: Off
Number of isotope peaks used for i-FIT = 2

Monoisotopic Mass, Even Electron Ions
152 formula(e) evaluated with 2 results within limits (all results (up to 1000) for each mass)
Elements Used:
C: 0-400 H: 0-1000 N: 7-7 O: 1-1 35Cl: 0-1 37Cl: 0-1
1

KE267

0130_1 38 (1.380) Cm (38:39-1x10.000)

30-Jan-2018
14:21:49
1: TOF MS ES+
9.14e+003

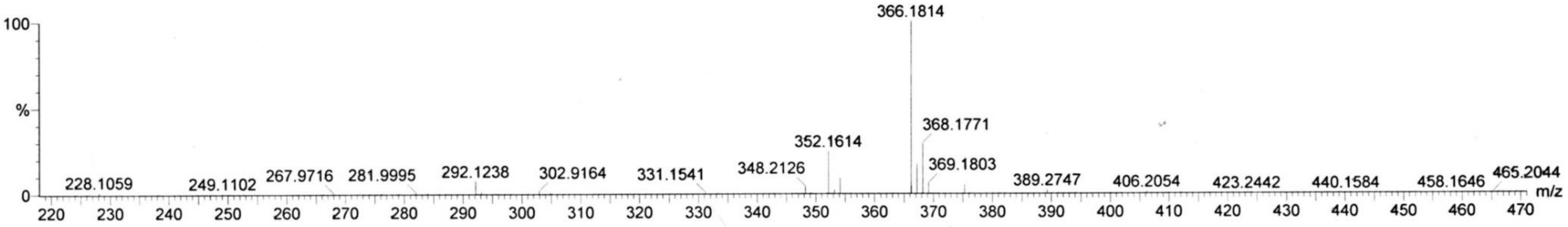

Minimum:	25.00				-1000.0			
Maximum:	100.00		5.0	30.0	1000.0			

Mass	RA	Calc. Mass	mDa	PPM	DBE	i-FIT	i-FIT (Norm)	Formula
366.1814	100.00	366.1809	0.5	1.4	7.5	46.0	0.0	C16 H25 N7 O 35Cl
368.1771	29.15	368.1780	-0.9	-2.4	7.5	42.6	0.0	C16 H25 N7 O 37Cl

Figure S4. ESI-HRMS spectrum of reaction adduct of **AIL 1** with ethanol gas

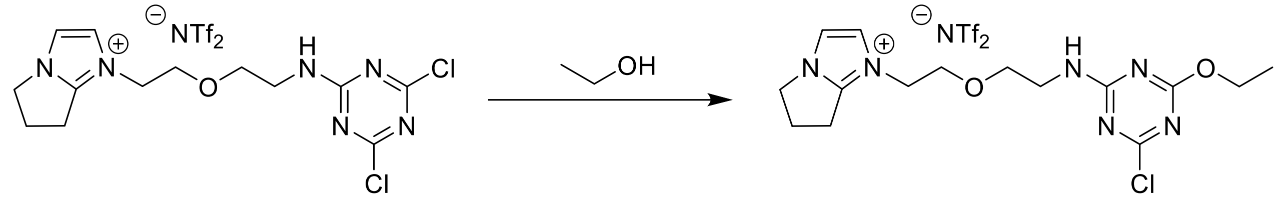

ESI-HRMS m/z $[M]^+$ calcd for $C_{15}H_{22}ClN_6O_2$ 353.1487, found 353.1479 ($[M]^+$, 100%), 355.1454 ($[M+2]^+$, 33%).

Spectrum

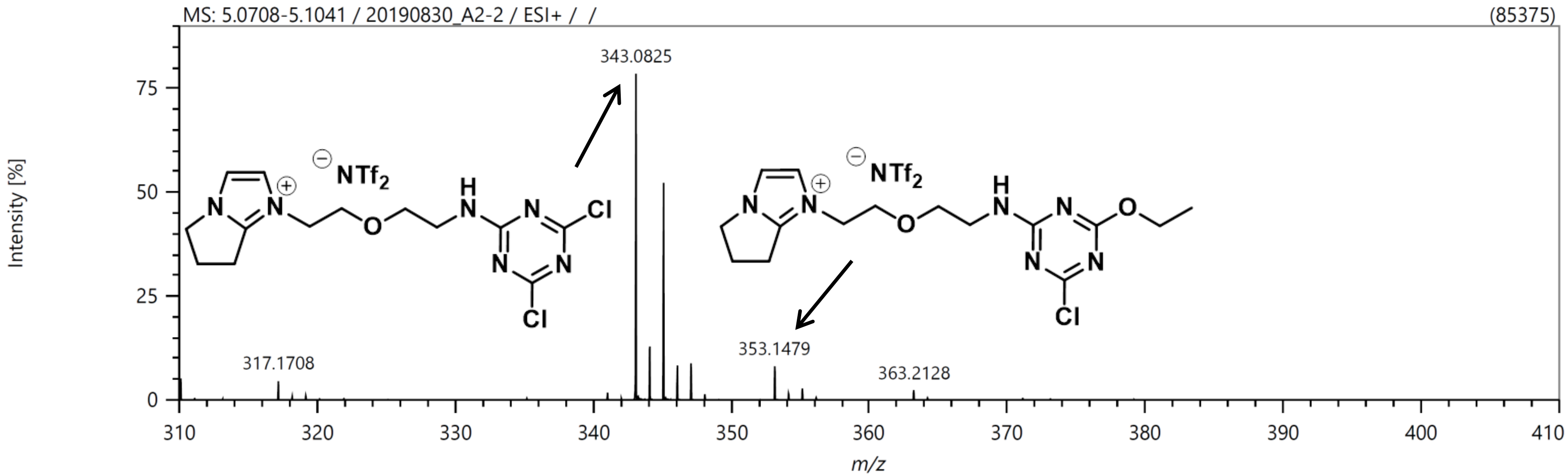

Elemental Composition

Parameters	
Tolerance:	±100.00 ppm
Electron:	Odd/Even
Charge:	+1
DBE:	-1.5 - 999.0

Elements Set 1:

Symbol	C	H	O	Na	N	Si	Cl	S
Min	0	0	2	0	6	0	1	0
Max	400	1000	2	0	6	0	1	0

Results

Mass	Formula	Calculated Mass	Mass Difference [mDa]	Mass Difference [ppm]	DBE
353.14790	C15 H22 N6 O2 Cl	353.14873	-0.83	-2.36	7.5

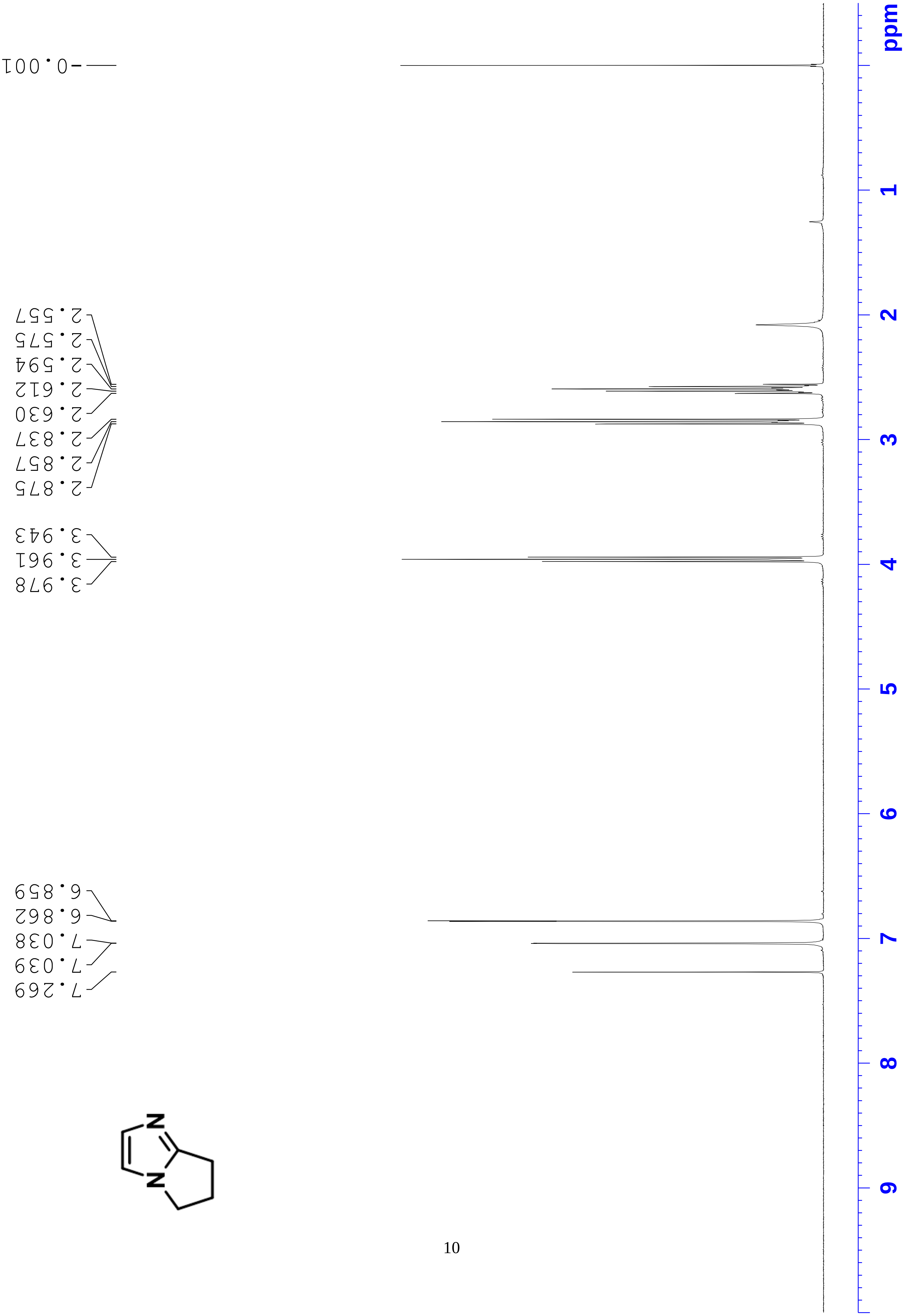
ppm
1
2
3
4
5
6
7
8
9
-0.001
2.557
2.575
2.594
2.612
2.630
2.837
2.857
2.875
3.943
3.961
3.978
6.859
6.862
7.038
7.039
7.269
N
N

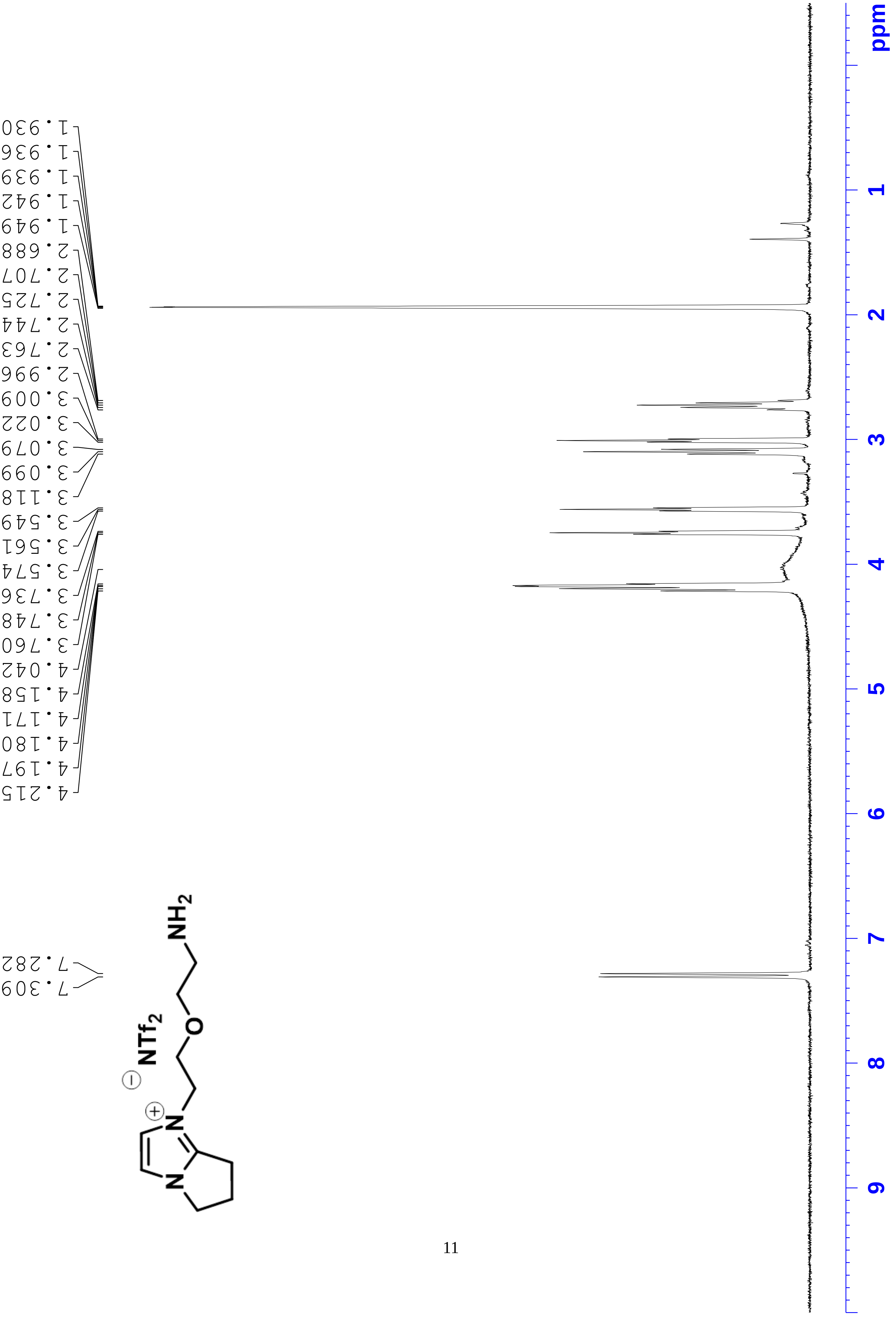
1.930
1.936
1.939
1.942
1.949
2.688
2.707
2.725
2.744
2.763
2.996
3.009
3.022
3.079
3.099
3.118
3.549
3.561
3.574
3.736
3.748
3.760
4.042
4.158
4.171
4.180
4.197
4.215
7.282
7.309
NTf2
NH2
ppm
1
2
3
4
5
6
7
8
9

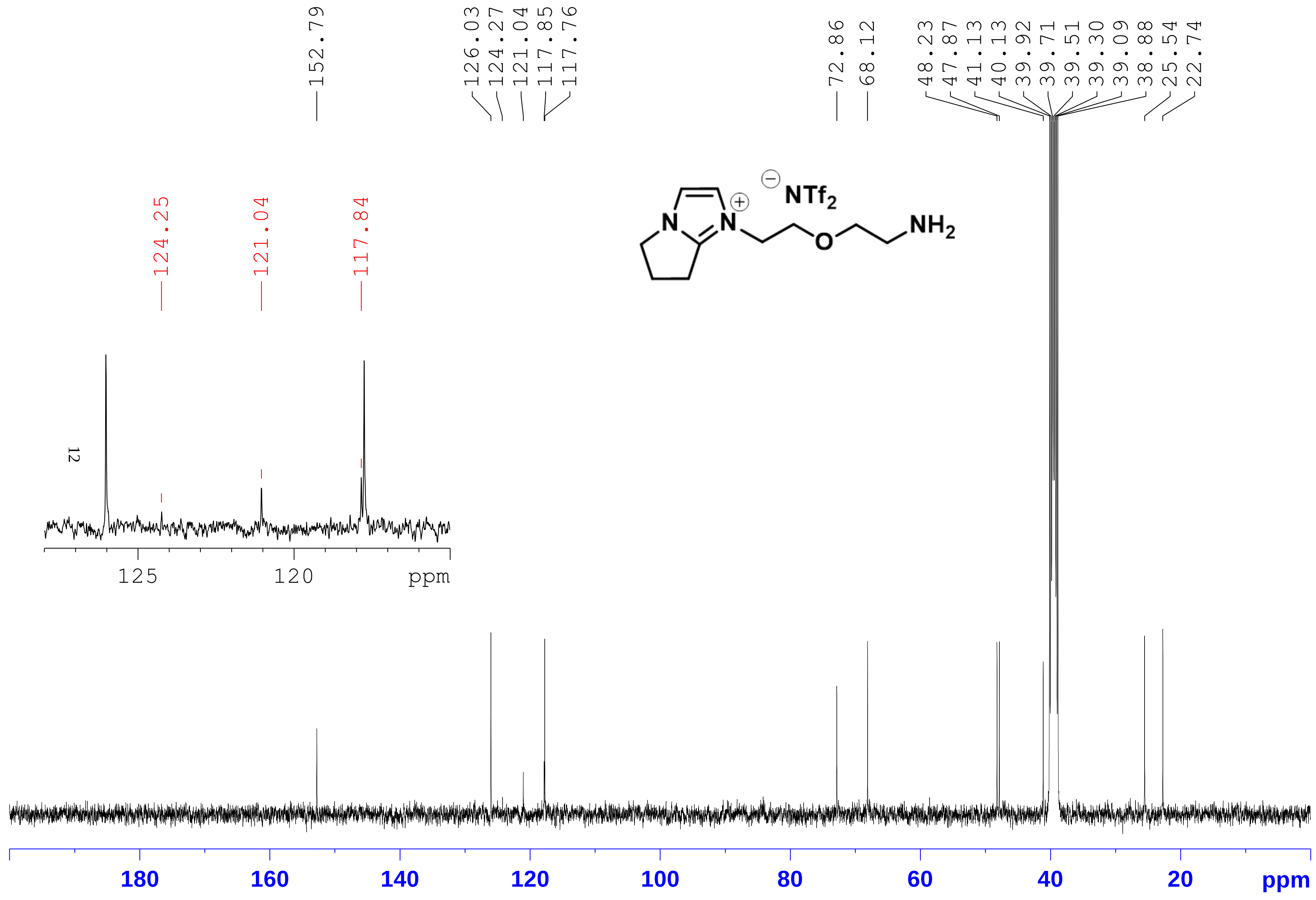
152.79
126.03
124.27
121.04
117.85
117.76
72.86
68.12
48.23
47.87
41.13
40.13
39.92
39.71
39.51
39.30
39.09
38.88
25.54
22.74
NTf2
NH2
124.25
121.04
117.84
125
120
ppm
180
160
140
120
100
80
60
40
20
ppm

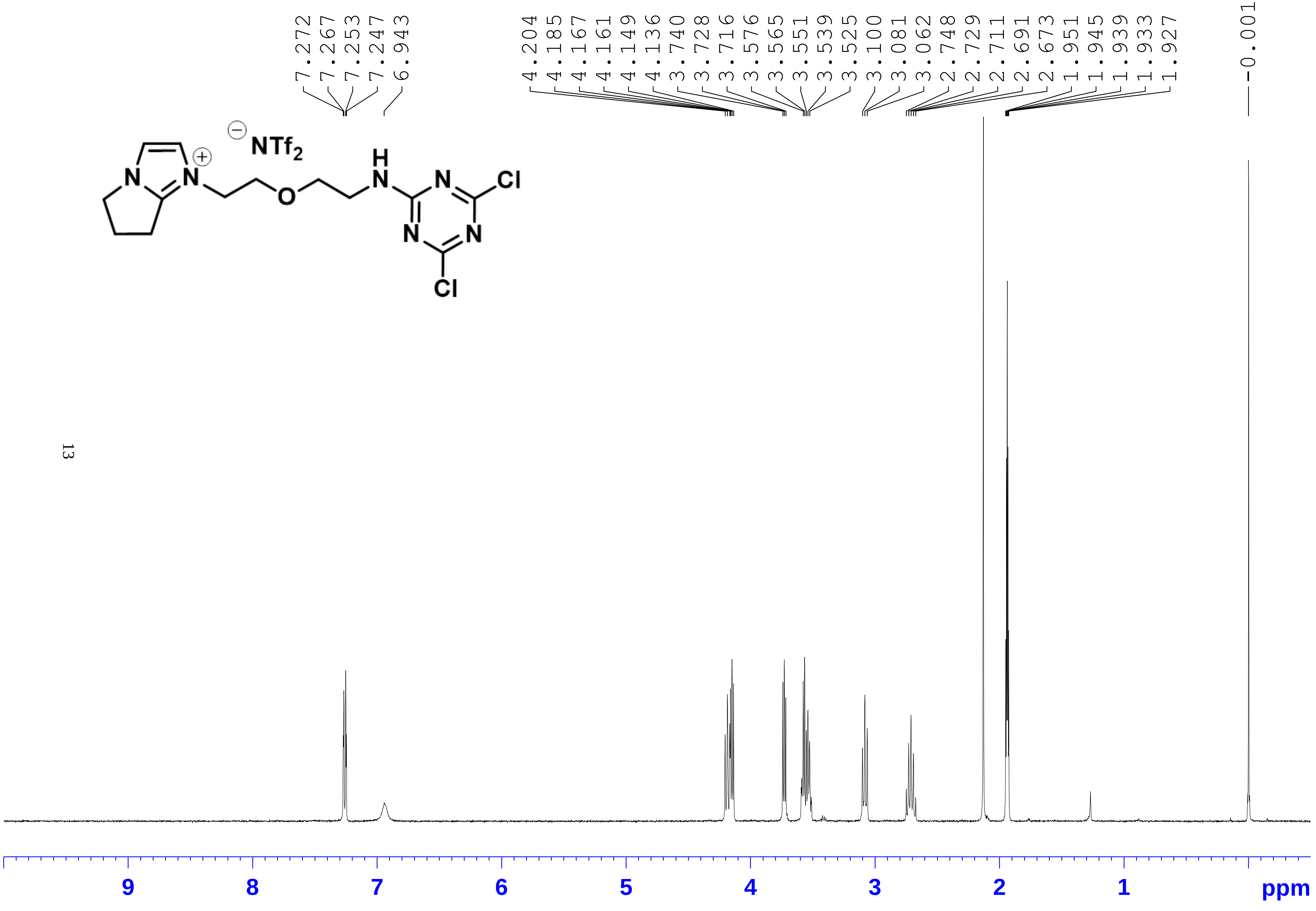
7.272
7.267
7.253
7.247
6.943
4.204
4.185
4.167
4.161
4.149
4.136
3.740
3.728
3.716
3.576
3.565
3.551
3.539
3.525
3.100
3.081
3.062
2.748
2.729
2.711
2.691
2.673
1.951
1.945
1.939
1.933
1.927
-0.001
NTf2
N
N
H
N
O
Cl
Cl
9
8
7
6
5
4
3
2
1
ppm

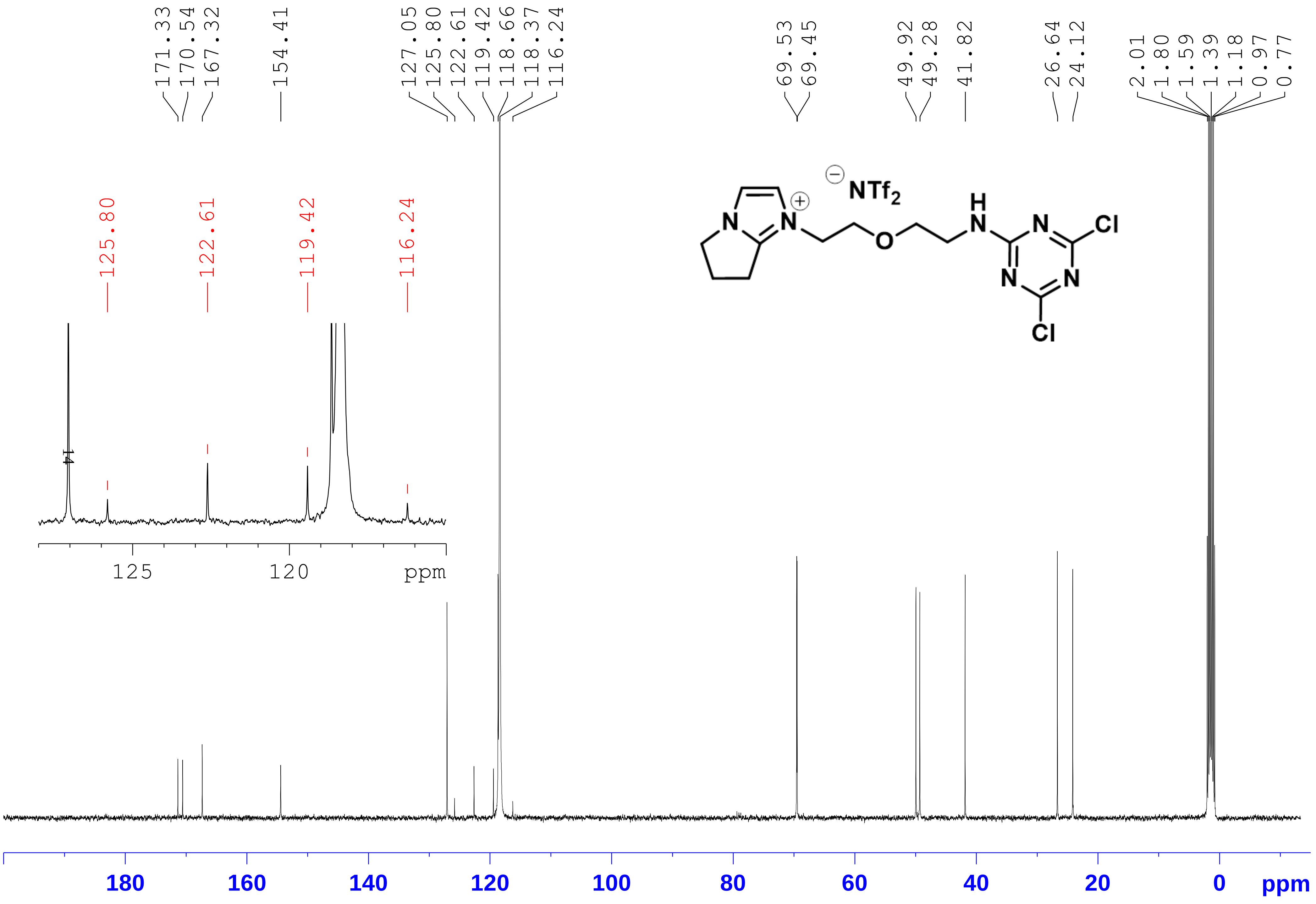
171.33
170.54
167.32
154.41
127.05
125.80
122.61
119.42
118.66
118.37
116.24
69.53
69.45
49.92
49.28
41.82
26.64
24.12
2.01
1.80
1.59
1.39
1.18
0.97
0.77
NTf2
N
O
H
Cl
125.80
122.61
119.42
116.24
125
120
ppm
180
160
140
120
100
80
60
40
20
0
ppm

Elemental Composition Report

Multiple Mass Analysis: 2 mass(es) processed

Tolerance = 5.0 PPM / DBE: min = -1000.0, max = 1000.0
Element prediction: Off
Number of isotope peaks used for i-FIT = 2

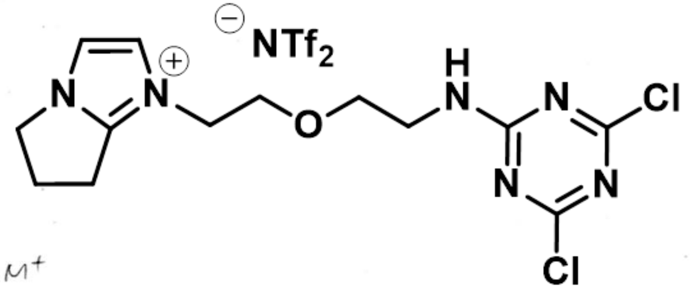

Monoisotopic Mass, Even Electron Ions
270 formula(e) evaluated with 2 results within limits (all results (up to 1000) for each mass)
Elements Used:
C: 0-400 H: 0-1000 N: 6-6 O: 1-1 35Cl: 0-2 37Cl: 0-2
2

0109_2 35 (1.264) Cm (35:36-1x10.000)

09-Jan-2018
15:14:15
1: TOF MS ES+
2.60e+004

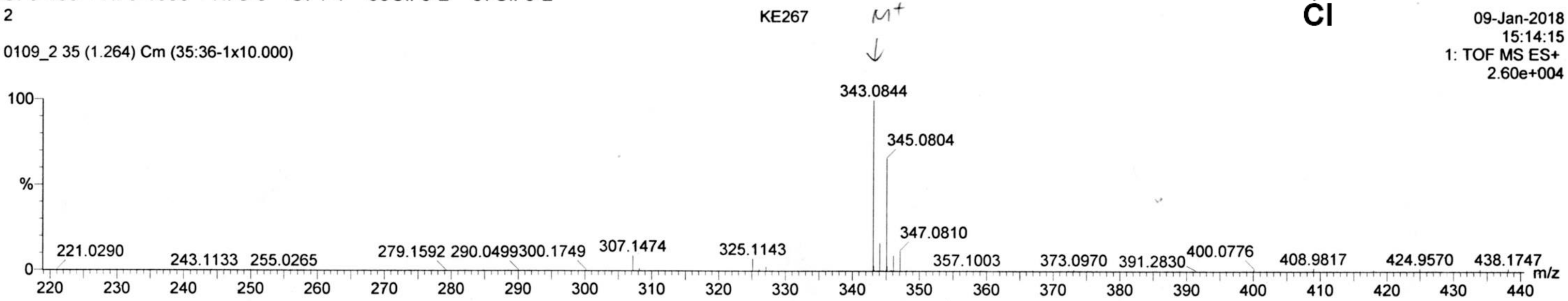

	Mass	RA	Calc. Mass	mDa	PPM	DBE	i-FIT	i-FIT (Norm)	Formula
Minimum:		50.00				-1000.0			
Maximum:		100.00		5.0	5.0	1000.0			
	343.0844	100.00	343.0841	0.3	0.9	7.5	150.9	0.0	C13 H17 N6 O 35Cl2
	345.0804	66.15	345.0811	-0.7	-2.0	7.5	108.4	0.0	C13 H17 N6 O 35Cl 37Cl

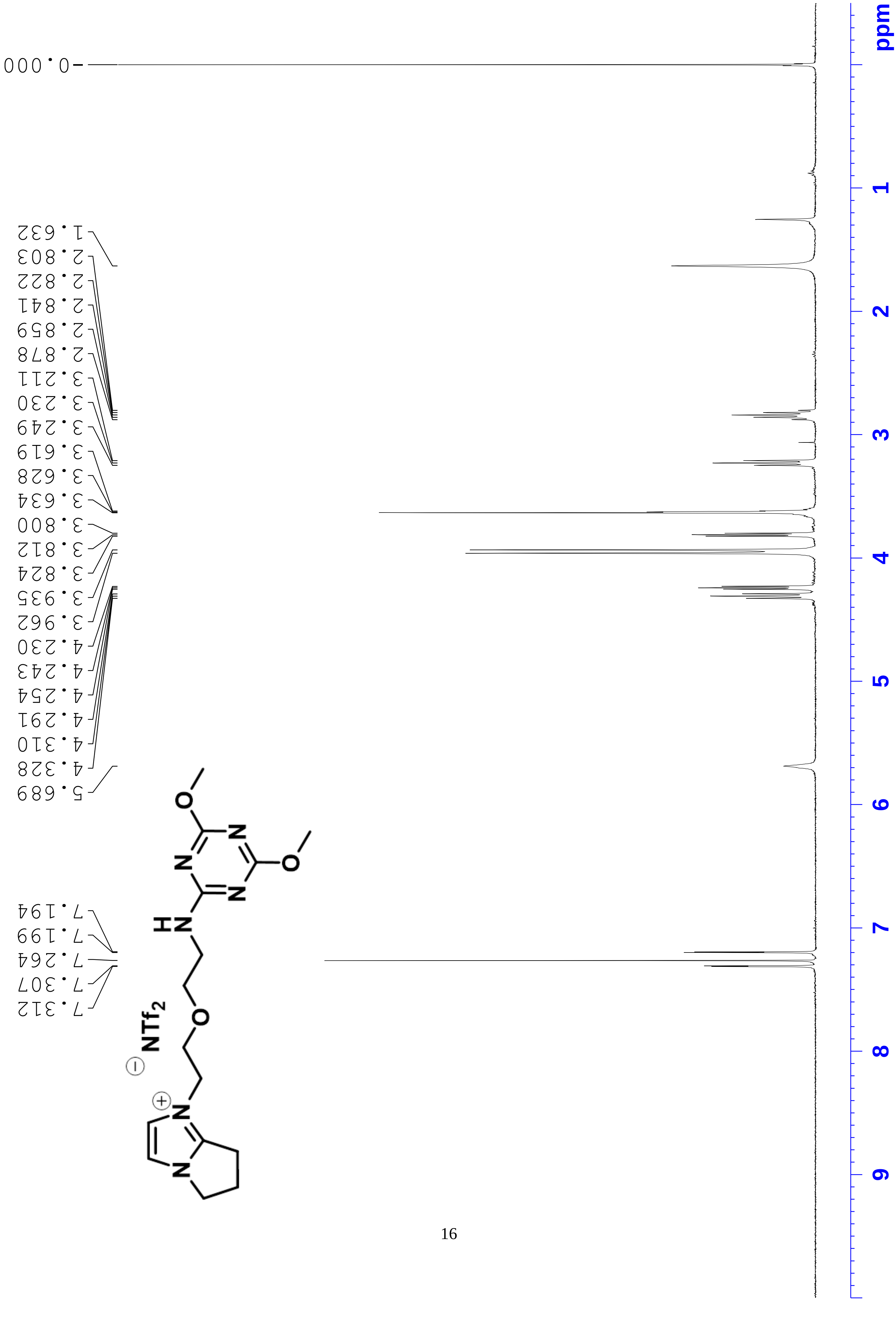
0.000
1.632
2.803
2.822
2.841
2.859
2.878
3.211
3.230
3.249
3.619
3.628
3.634
3.800
3.812
3.824
3.935
3.962
4.230
4.243
4.254
4.291
4.310
4.328
5.689
7.194
7.199
7.264
7.307
7.312
ppm
1
2
3
4
5
6
7
8
9
NTf2

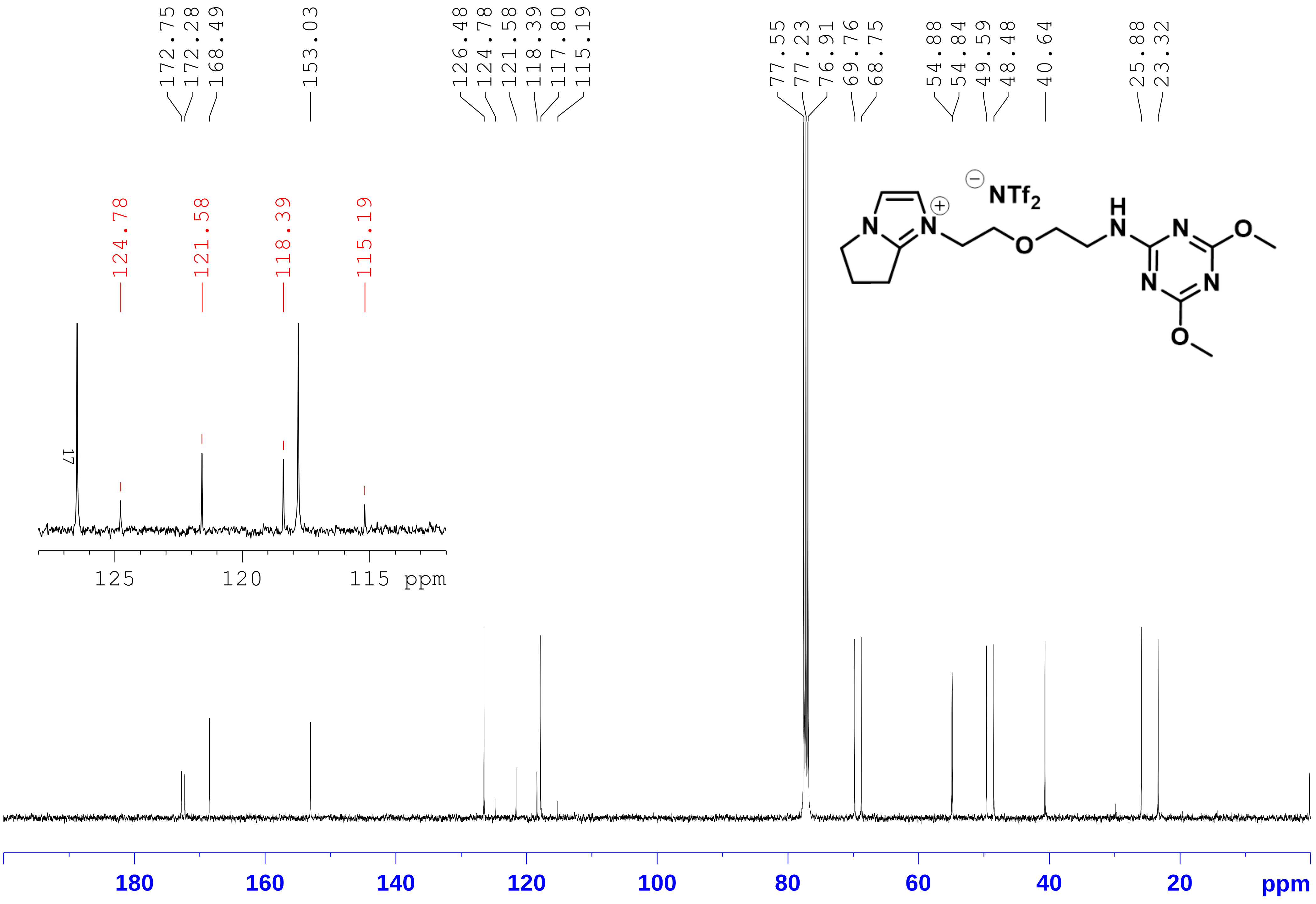
172.75
172.28
168.49
153.03
126.48
124.78
121.58
118.39
117.80
115.19
77.55
77.23
76.91
69.76
68.75
54.88
54.84
49.59
48.48
40.64
25.88
23.32
NTf2
124.78
121.58
118.39
115.19
125
120
115 ppm
180
160
140
120
100
80
60
40
20
ppm

Elemental Composition Report

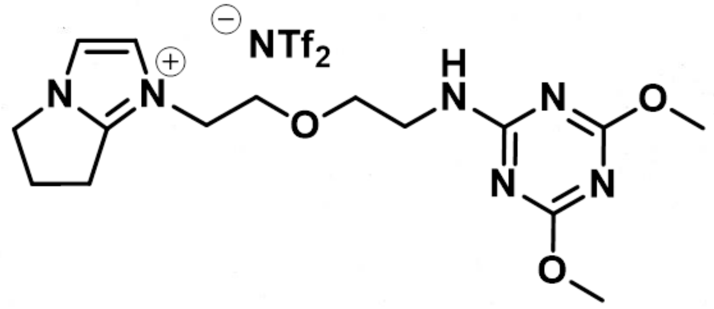

Single Mass Analysis

Tolerance = 30.0 PPM / DBE: min = -1000.0, max = 1000.0
Element prediction: Off
Number of isotope peaks used for i-FIT = 2

Monoisotopic Mass, Even Electron Ions
18 formula(e) evaluated with 1 results within limits (all results (up to 1000) for each mass)
Elements Used:
C: 0-400 H: 0-1000 N: 6-6 O: 3-3
7

KE267

15-Oct-2018
16:26:19
1: TOF MS ES+
5.51e+005

1015_7_2 12 (0.805) Cm (12:13-100x10.000)

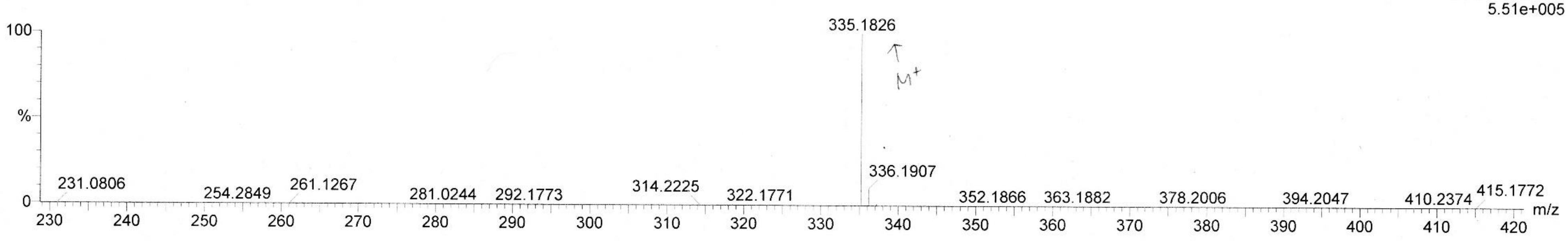

Minimum:				-1000.0			
Maximum:		5.0	30.0	1000.0			

Mass	Calc. Mass	mDa	PPM	DBE	i-FIT	i-FIT (Norm)	Formula
335.1826	335.1832	-0.6	-1.8	7.5	64.4	0.0	C15 H23 N6 O3
